# The Progression of Cell Death Affects the Rejection of Allogeneic Tumors in Immune-Competent Mice – Implications for Cancer Therapy

**DOI:** 10.3389/fimmu.2014.00560

**Published:** 2014-11-11

**Authors:** Ricardo A. Chaurio, Luis E. Muñoz, Christian Maueröder, Christina Janko, Thomas Harrer, Barbara G. Fürnrohr, Michael Niederweis, Rostyslav Bilyy, Georg Schett, Martin Herrmann, Christian Berens

**Affiliations:** ^1^Department of Internal Medicine 3, Friedrich-Alexander University of Erlangen-Nuremberg, Erlangen, Germany; ^2^Department of Biology, Friedrich-Alexander University of Erlangen-Nuremberg, Erlangen, Germany; ^3^Department of Otorhinolaryngology, Head and Neck Surgery, Section for Experimental Oncology and Nanomedicine (SEON), University Hospital Erlangen, Erlangen, Germany; ^4^Division of Molecular Immunology, Department of Internal Medicine 3, Friedrich-Alexander University of Erlangen-Nuremberg, Erlangen, Germany; ^5^Division of Biological Chemistry, Medical University Innsbruck, Innsbruck, Austria; ^6^Department of Microbiology and Biochemistry, University of Alabama at Birmingham, Birmingham, AL, USA; ^7^Institute of Cell Biology, National Academy of Sciences of Ukraine, Lviv, Ukraine

**Keywords:** immunogenicity, apoptosis, cancer, ROS, caspase-3, tBid, necrosis, DAMPs

## Abstract

Large amounts of dead and dying cells are produced during cancer therapy and allograft rejection. Depending on the death pathway and stimuli involved, dying cells exhibit diverse features, resulting in defined physiological consequences for the host. It is not fully understood how dying and dead cells modulate the immune response of the host. To address this problem, different death stimuli were studied in B16F10 melanoma cells by regulated inducible transgene expression of the pro-apoptotic active forms of caspase-3 (revCasp-3), Bid (tBid), and the *Mycobacterium tuberculosis*-necrosis inducing toxin (CpnT_CTD_). The immune outcome elicited for each death stimulus was assessed by evaluating the allograft rejection of melanoma tumors implanted subcutaneously in BALB/c mice immunized with dying cells. Expression of all proteins efficiently killed cells *in vitro* (>90%) and displayed distinctive morphological and physiological features as assessed by multiparametric flow cytometry analysis. BALB/c mice immunized with allogeneic dying melanoma cells expressing revCasp-3 or CpnT_CTD_ showed strong rejection of the allogeneic challenge. In contrast, mice immunized with cells dying either after expression of tBid or irradiation with UVB did not, suggesting an immunologically silent cell death. Surprisingly, immunogenic cell death induced by expression of revCasp-3 or CpnT_CTD_ correlated with elevated intracellular reactive oxygen species (ROS) levels at the time point of immunization. Conversely, early mitochondrial dysfunction induced by tBid expression or UVB irradiation accounted for the absence of intracellular ROS accumulation at the time point of immunization. Although ROS inhibition *in vitro* was not sufficient to abrogate the immunogenicity in our allo-immunization model, we suggest that the point of ROS generation and its intracellular accumulation may be an important factor for its role as damage associated molecular pattern in the development of allogeneic responses.

## Introduction

The appearance of cell death during disease therapy is a two-pronged sword. On one hand, cell death is desirable during cancer treatment in order to control malignant cell growth in the patient. On the other hand, excessive cell death should be avoided during transplantation to allow the grafted cells to survive in a foreign host. In the latter case, a considerable number of stresses are involved and several kinds of injuries may additionally compromise tissue viability, leading to progressive graft dysfunction and, eventually, also to graft loss (1, 2).

Furthermore, the mechanism and type of cell death might profoundly affect the reaction of the host toward surviving cells, making the situation more complex. Apoptosis, necrosis, autophagy, necroptosis, and other processes have been reported as common cell death mechanisms observed *in vivo* during therapies. However, how these types of cell death modulate interactions of the dying and dead cells with the immune system remains elusive. Depending on the immune response elicited, it is possible to distinguish between cases of cell death able to induce immunogenicity (immunogenic cell death) and those inducing immune tolerance or unresponsiveness (tolerogenic/silent cell death) (3, 4). Dying cells can exhibit completely different characteristics and immunological features. To understand these differences, an accurate characterization of the features, types, and phases of cell death is required.

The latter has become especially important in the context of diseases like cancer where conventional treatments (e.g., radiation and chemotherapy) are based on the massive induction of tumor cell death. In such cases, the immune system is prone to be decisive for tumor fate. Because the guidelines for drug screening in antineoplastic therapies require evaluation of human tumors xenotransplanted into immune-compromised mice (5), the role of the immune system has been neglected (6), making studies focused on the interplay between immune system and dying cells necessary. Modern anti-cancer therapies aim at inducing immunogenic cancer cell death. However, there are a plethora of factors involved in this process that have to be revisited and reassessed carefully. These include intrinsic cell immunogenicity, the nature of the initial death stimulus, the type of damage associated molecular patterns (DAMPs) released, the clearance capacity of the affected tissue for dying and dead cells, and the respective death pathway. Considering the large number of cytotoxic drugs currently used in the treatment of neoplastic diseases, much information is missing to predict the anti-tumor response of the host reliably.

In this study, we showed how different mechanisms and types of cell death, induced by different stimuli, affect the outcome of allogeneic tumor transplants in BALB/c immune-competent mice. Additionally, a morpho-physiological characterization of dying and dead cells, based on a multiparametric flow cytometry analysis, was assessed. A murine allograft model allowed evaluation of the immune response *in vivo*. The results of this work may have important implications for both cancer therapy and procedures for experimental allotransplantation.

## Materials and Methods

### Reagents and molecular probes

Dulbecco’s modified eagle’s medium (DMEM), fetal bovine serum (FBS), G418, penicillin–streptomycin, and glutamine were from Gibco-Invitrogen. Recombinant chicken annexin A5 (AxA5) was purchased from Responsif. The FluoroTag fluorescein isothiocyanate (FITC) conjugation kit was from Sigma-Aldrich, the 1,10,3,3,30,30-hexamethylindodicarbocyanine iodide dye [DiIC1(5)], Hoechst 33342, Lipofectamine™ 2000, and puromycin dihydrochloride were from Invitrogen. Propidium iodide (PI) was obtained from Amersham Biosciences. Doxycycline hydrochloride and trypsin–EDTA solution were purchased from Sigma-Aldrich. Ringer’s solution was from Delta Select. The caspase3 inhibitor z-DEVD-fmk, the caspase-9 inhibitor Ac-LEHD-cmk, and the general caspase inhibitor z-VAD-fmk were purchased from Bachem.

### Cell lines and culture conditions

The C57BL/6 mouse-derived melanoma cell line B16F10 bearing the haplotype H2b was purchased from ATCC (#CRL-6475) and propagated in DMEM supplemented with 10% FBS and penicillin–streptomycin (D10) at 37°C in a 5% CO_2_ atmosphere.

For the morpho-physiological characterization by flow cytometry, B16F10 cells were cultured in 24-well plates at 100,000 cells/2 ml D10 and harvested at the time points indicated. Harvesting was performed as follows: supernatants containing dead cells were harvested into polypropylene tubes. A trypsin–EDTA solution was added to the wells for 10–15 min at room temperature to detach the remaining adherent cells. Thus, detached cells were collected by adding D10 and combined with their corresponding supernatant fraction. Finally, cells were centrifuged at 300 g for 5 min, resuspended in 500 μl D10 medium.

### Generation of stable Tet-controlled suicide cell lines

**Figure 1:**
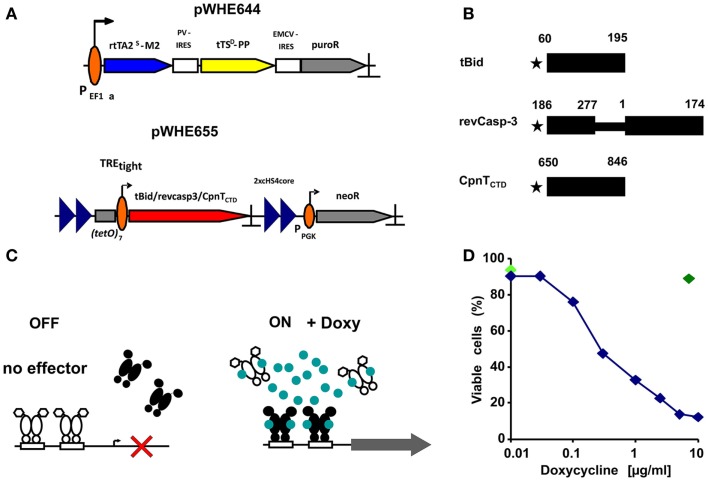
**Conditional expression of death inducing proteins**. **(A)** Schematic overview of the constructs used to establish the regulatory system. The vector pWHE644 represents the regulator construct. A human EF1α promoter constitutively transcribes a tricistronic mRNA. This mRNA contains the reverse transactivator rtTA2^S^-M2 (blue arrow), the transsilencer tTS^D^-PP (yellow arrow), and a selection marker (puromycin resistance; gray arrow). Translation of the latter two genes is mediated by internal ribosome entry sites (IRES; open boxes) from polio-virus (PV) and encephalomyocarditis- virus (EMCV). The vector pWHE655 contains the response unit used for stable transfections. It features the target gene (red arrow) driven by the Tet-responsive promoter TRE_tight_ (open box, broken arrow) and flanked by two repeats each of a 250 bp sequence from the chicken HS4 insulator (blue triangles). A murine phosphoglycerate kinase 1 promoter (PGK; broken arrow) drives expression of a gene mediating G418-resistance. PolyA sites in all vectors are marked by a “ ⊥.” **(B)** Schematic representation of the cytotoxic test proteins. The residues that border the active domains expressed in the experiment are indicated above their respective closed box. A methionine added to allow translation is represented by a star. **(C)** Schematic overview of the regulatory system. In the OFF-State, a transsilencer (white) binds to the minimal promoter (open boxes, broken arrow) and actively suppresses transcription (cross). In the ON-State, doxycycline (blue circles) binds to both transsilencer and reverse transactivator (black). The former dissociates from, the latter binds to the minimal promoter and activates transcription (gray arrow). **(D)** Response of the regulatory system to different doxycycline concentrations. The B16F10-tBid transfected cell line was incubated for 24 h with various concentrations of doxycycline and mortality was measured, shown for one representative experiment out of three performed. Concentrations between 5 and 10 μg/ml showed the highest extent of cell death. An additional control at 10 μg/ml Doxy with the parental stably transfected cell line B16F10-644 was included to discard doxycycline toxicity at higher concentrations as cause of cell death (dark green diamond). Cell viability at time point “0” is shown as light green diamond.

The cell lines B16F10-644, B16F10-tBid, and B16F10-revCasp-3 have been described (7). The parental cell line B16F10-644 was transfected with the *Ahd*I-linearized plasmid pWHE655TREtight-CpnT_CTD_, carrying the C-terminal domain of the channel protein with necrosis inducing toxin (CpnT_CTD_) characterized in *Mycobacterium tuberculosis* (8) (Figures 1A–C), and stable transfectants were selected by limited dilution in the presence of 1500 μg/ml G418. Individual subclones were cultured in 48-well plates and tested for cell death with AxA5/PI staining by FACS after 24 h of doxycycline (1 μg/ml) addition. One out of several positive clones was chosen for further experiments and named B16F10-CpnT_CTD_.

### Multi-parameter classification of cell death by flow cytometry

**Figure 2:**
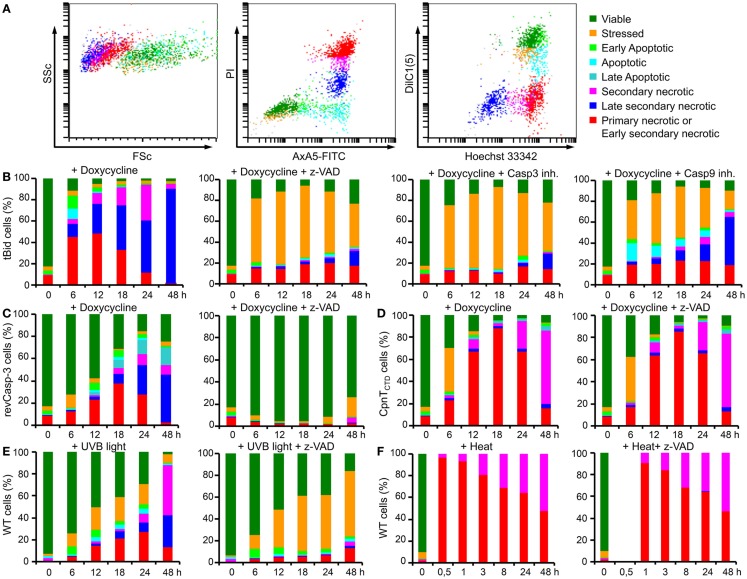
**Six parameter classification by flow cytometry of the cell death phenotype of dying and dead B16F10 cells**. Cell death analysis is based on morphological features (FSc and SSc), on the exposure of PS (annexin A5-FITC) and plasma membrane ion selectivity (PI), on the mitochondrial membrane potential [DiIC1(5)] and on nuclear DNA content (Hoechst 33342) detected by flow cytometry. Note: after proper gating, up to eight physiologically different subpopulations can be recorded. Dot plots exemplarily show B16F10-revCasp-3 cells after 18 h of doxycycline (5 μg/ml) treatment **(A)**. Rapid cell death occurred after 6 h in tBid-expressing cells and more than 95% cell death was observed after 24 h. In the presence of various caspase inhibitors [z-VAD-fmk, z-DEVD-fmk (caspase-3 inhibitor) and Ac-LEHD-cmk (caspase-9 inhibitor); all 50 μM], a significant increase in the stressed cell fraction displaying low-mitochondrial potential was observed **(B)**. Expression of revCasp-3 in B16F10 cells induced cell death after 24 h in more than 80% of the cells. z-VAD-fmk (50 μM) completely inhibited doxycycline-driven apoptosis. Note: stressed cells do not arise in this type of cell death induction **(C)**. Expression of CpnT_CTD_ induced cell death in more than 90% of the cells after 18 h. Note: primary necrosis was the most common type of cell death observed and death occurred independently of caspase activity (50 μM z-VAD-fmk) **(D)**. Lethal UVB irradiation (240 mJ/cm^2^) of parental B16F10 cells causes a rather slow progressing kind of cell death. Note: in the presence of z-VAD-fmk (50 μM), a significant increase of the stressed cell fraction displaying low-mitochondrial potential was observed **(E)**. Heat shock (56°C, 30 min) caused immediate necrosis in 100% of cells independent of caspase activity **(F)**. Displayed are the mean values from three independent experiments of relative percentages of each cell phenotype during 48 h of culture **(B–F)**.

The cell death characterization method analyzing size, granularity, PS exposure, plasma membrane integrity, mitochondrial membrane potential, and DNA content in a one-tube-measurement has been thoroughly described elsewhere (9). This method classifies eight different phases of cell death. Briefly, the harvested cells were incubated for 30 min at room temperature with 400 μl of freshly prepared 4-color staining solution [1.8 μg/ml AxA5-FITC, 100 ng/ml PI, 10 nM DiIC1(5), 1 ng/ml Hoechst 33342] in Ringer’s solution and subsequently analyzed. Flow cytometry was performed with a Gallios cytofluorometer (Beckman Coulter, Fullerton, CA, USA). Excitation of FITC and PI was at 488 nm, the FITC fluorescence was detected with the FL1 sensor (525/38 nm BP), the PI fluorescence with the FL3 sensor (620/30 nm BP), the DiIC1(5) fluorescence was excited at 638 nm and detected with the FL6 sensor (675/20 nm BP), and the Hoechst 33342 fluorescence was excited at 405 nm and detected with the FL9 sensor (430/40 nm BP). Electronic compensation was applied to reduce bleed-through fluorescence. Data analysis was performed with Kaluza software version 2.0 (Beckman Coulter, Fullerton, CA, USA). Cells were classified according to their location in the forward scatter (FSc; size) vs. side scatter (SSc; granularity) dot plot and their staining patterns in the FL1 vs. FL3 and FL6 vs. FL9 dot plots (Figure 2A).

### Cell death induction

Cell death was induced by (1) doxycycline *in vitro* (5–10 μg/ml) for expression of the cell death inducing proteins tBid, revCasp-3, and CpnT_CTD_. (2) Irradiation with ultraviolet light type B (UVB) at 1.5 mJ/cm^2^/s. (3) By heat shock (56°C for 30 min).

### Measurement of intracellular reactive oxygen species

Intracellular reactive oxygen species (ROS) levels were assessed using the redox-sensitive dye 2,7-dichlorofluorescein diacetate (C-DCFH-DA). At the time points indicated, dying B16F10 cells were harvested, incubated for 30 min with C-DCFH-DA (10 μM) at 37°C in protein-free medium (D0) in the dark. Cells were then washed with D10 by centrifugation, co-stained with PI, and analyzed by flow cytometry. Only PI-negative cells were analyzed and ROS levels were presented as the mean fluorescence intensity in the FL1 channel (MFI-FL1). The anti-oxidants *N*-acetylcysteine (NAC) and 2-(2,2,6,6-Tetramethylpiperidin-1-oxyl-4-ylamino)-2-oxoethyl) triphenylphosphonium chloride (mitoTEMPO) were used at 100 μM as indicated.

### Allogeneic tumor growth model and immunizations

The allogeneic tumor growth model consisted of the host mouse (BALB/c, MHC haplotype H2d) and B16F10 melanoma cells derived from C57BL/6 mice (MHC haplotype H2b). Specified amounts of viable cells or dead/dying cells were subcutaneously (s.c.) implanted in 500 μl Ringer’s solution in the right flank using a syringe with a 25 G needle. The growth of solid melanoma tumors was registered by direct measurement of width, height, and depth of the black subcutaneous protuberance with a caliper for up to a maximum of 40 days. In accordance with the guidelines for the welfare of animals in experimental neoplasia, the animal was sacrificed if the mouse tumor volume exceeded more than 10% of the host’s body weight.

In order to evaluate the immune response against implanted cells, mice were challenged s.c. with 2 million viable cells in the opposite flank, after immunization as indicated. The use of the syngeneic host (C57BL/6) as recipient of B16F10 melanoma cell lines was ruled out because of the aggressiveness of the B16F10 cells, which may cause discomfort and premature death of the animal. Mice were purchased from Charles River Laboratories International, Inc., and kept on a standard diet with drinking water available *ad libitum*. Experiments were conducted according to the European principles and local guidelines for care and use of laboratory animals at the Institute of Cell Biology, National Academy of Sciences of Ukraine, Lviv (10–12).

### Statistical analysis

The software package GraphPad Prism 5.0 was used for graphics and statistical tests. For comparisons between control and experimental groups, Mann–Whitney *U* test or two way ANOVA tests were employed as appropriate. Statistical significance was assumed if *p* < 0.05.

## Results

### Stable Tet-controlled mouse melanoma B16F10 suicide cell lines

Subcloned transfected mouse melanoma B16F10 cell lines were tested for the response of the regulatory system to different doxycycline (doxy) concentrations after 24 h. Mortality rates were calculated by annexin A5/PI staining and flow cytometry. Concentrations around 10 μg/ml doxy showed the highest degree of cell death (Figure 1D).

### Cell death induced by expression of cytotoxic proteins in B16F10 cells and morpho-physiological classification of dead and dying tumor cells by flow cytometry

Since dying cells may exhibit various biological features that modulate the immune response, it is necessary to accurately characterize the phenotypes and phases of cell death. Employing a six parameter protocol to characterize the morphological and physiological features of dying and dead cells, we identified eight different states of cell death in B16F10 melanoma cells: (1) viable; (2) stressed; (3) early apoptosis; (4) apoptosis; (5) late apoptosis; (6) secondary necrosis; (7) late secondary necrosis; and (8) primary necrosis or early secondary necrosis (Figure 2A) (9, 13). This method allows us to closely describe biological features of death in cell lines expressing cytotoxic proteins. In our test system, doxycycline relieves active repression of the promoter by the tetracycline-dependent transsilencer (tTS) and simultaneously induces binding of the reverse tetracycline-dependent transactivator (rtTA) leading to transgene expression of each death-inducing-protein (Figure 1C). Additionally, the classical death stimuli UVB irradiation and heat shock were also employed.

Activation of the suicide switch in B16F10-tBid cells with 5 μg/ml doxy was followed by very fast and efficient killing. After 6 h, up to 90% of the cells were dead. Approximately 30% of the cells displayed features typical for initial phases of apoptosis and the rest of the cells showed a necrotic phenotype, predominantly early secondary necrosis (Figure 2B). The role of caspases in the cell death induced by tBid in B16F10 cells was studied by adding the pan-caspase inhibitor z-VAD-fmk (50 μM). Considering the physiological stringency of this method, treatment with z-VAD-fmk did not change the fraction of viable cells treated with doxy. Interestingly, a significant increase in the fraction of stressed cells was observed for all times points. Stressed cells are defined as cells with conserved membrane asymmetry, membrane integrity, and cell morphology, but very low-mitochondrial membrane potential [DiIC1(5) low] (13). This fraction reached its maximum at 18 h post-induction with up to 80% of the cells displaying a stressed phenotype (Figure 2B). In this case, stressed cells can be considered to be in a “pre-mortal” state, induced by the expression of tBid and concomitant inhibition of caspases. Stressed cells are detected efficiently by the six parameter method described above. tBid death kinetics in the presence of the more specific caspase-3 and caspase-9 inhibitors were similar to those observed in the presence of z-VAD-fmk, and showed comparable amounts of dead cells (Figure 2B).

Expression of revCasp-3 in B16F10 cells induced cell death after 24 h, albeit more slowly than tBid, with more than 80% of the cells displaying dead phenotypes (Figure 2C). Early stages of apoptosis were observed at 12 and 18 h, while late apoptotic and secondary necrotic stages were more predominant at 24 and 48 h. As expected, cell death by expression of revCasp-3 was completely inhibited in the presence of z-VAD-fmk (Figure 2C).

Expression of the necrosis inducing protein CpnT_CTD_ in B16F10 cells led to cell death after 18 h with more than 80% of the cells displaying features of primary necrosis (Figure 2D). Early stages of apoptosis were not detected by the expression of this protein. In contrast to pro-apoptotic proteins, z-VAD-fmk did not affect the death phenotype induced by CpnT_CTD_, suggesting a caspase-independent type of cell death (Figure 2D).

It is important to note that cell death induced by doxycycline-controlled expression of the proteins tBid, revCasp-3, and CpnT_CTD_ did not kill all cells – some cells may have failed to respond to doxy. In agreement with this assumption, long-term culture of doxy-treated cells resulted in confluent growth of the surviving non-responder cells.

The parental cell line B16F10-644 was lethally irradiated with UVB (240 mJ/cm^2^). After 6 h, some stressed and early apoptotic cells were observed. After 12 h, stressed, apoptotic, and some forms of necrotic cells were present. Secondary necrotic cells increasingly appeared from 24 to 48 h. The presence of z-VAD-fmk produced an important increase in the fraction of stressed cells preserving the fraction of viable cells, as was also observed for cells expressing tBid (Figure 2E). UVB irradiation caused much slower death in comparison to that induced by the expression of tBid, revCasp-3, or CpnT_CTD_. Importantly, no surviving cells were observed even when the plates were cultured for 7 days after irradiation.

Cell death by heat shock was studied in the parental cell line B16F10-644. Cells were incubated at 56°C for 30 min in a water bath to induce abrupt membrane disruption. More than 98% of the cells became primary necrotic. No differences were observed by the addition of z-VAD-fmk (Figure 2F). In summary, the suicide switch system efficiently induced morpho-physiologically different forms of tumor cell death.

### Reactive oxygen species production in dying tumor cells

**Figure 3:**
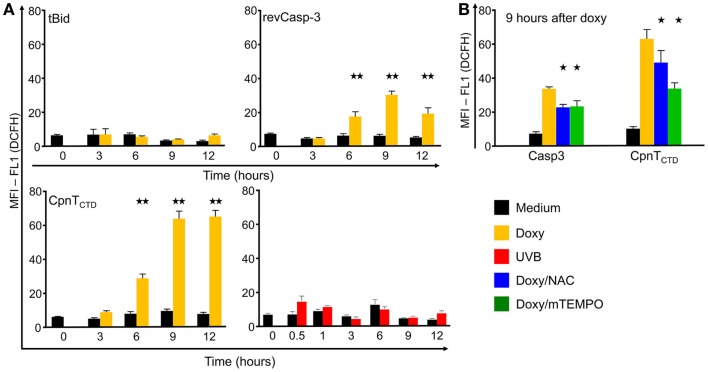
**Reactive oxygen species (ROS) production by dying B16F10 melanoma cells**. Cells were induced to die by conditional expression of the death proteins tBid, revCasp-3, and CpnT_CTD_ or by UVB irradiation, stained with the ROS sensor DCFH and with PI and analyzed by flow cytometry **(A)**. Inhibition of ROS production was performed by treatment with *N*-acetyl-cysteine (NAC) or mitoTEMPO, 100 μM, respectively, and recorded at 9 h after death induction **(B)**. Mean and SEM values of the mean fluorescence intensities of FL1 in viable cells (PI-negative) are displayed for different time points. At least three independent experiments were performed (Two and one stars indicate statistical significance at the *p* < 0.001 and *p* < 0.05 levels, respectively).

In order to further characterize the aforementioned forms of cell death, we measured the ability of the dying cells to produce free radicals. After addition of doxy to B16F10-revCasp-3 and B16F10-CpnT_CTD_ cells, a significant, five to sixfold, increase in the production of ROS was observed after 6 h and reached its maximum at 9 h in both cell lines. Interestingly, dying B16F10-CpnT_CTD_ cells showed the highest accumulation of ROS (12-fold) before disruption of the plasma membranes occurred (Figure 3). This response was compromised by the addition of the anti-oxidants *N*-acetylcysteine (NAC) and 2-(2,2,6,6-Tetramethylpiperidin-1-oxyl-4-ylamino)-2-oxoethyl) triphenylphosphonium chloride (mitoTEMPO). In contrast, B16F10-tBid expressing and UVB-irradiated dying cells did not produce significant amounts of ROS up to 12 h after death induction.

### Growth of B16F10 cells in the allogeneic BALB/C host and concomitant immunity

**Figure 4:**
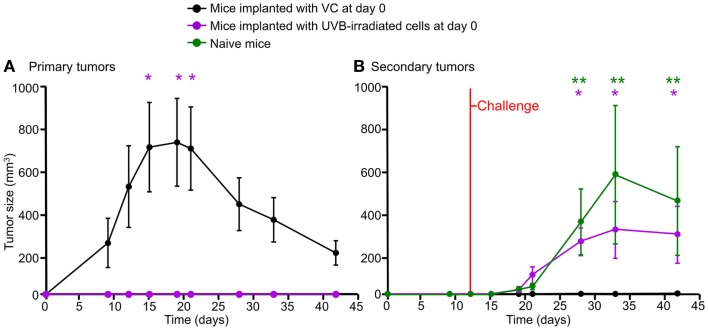
**Growth of B16F10 melanoma cells in the allogeneic host and concomitant immunity**. Four million viable B16F10 cells (VC) were implanted s.c. in the right flank of BALB/c mice. Mice developed tumors reaching their maximum size after 2–3 weeks, followed by rejection [**(A)**, black line]. Mice implanted with 4 million UVB-irradiated cells did not develop primary tumors [**(A)**, purple line]. After challenge with 2 million viable cells s.c. on the left flank, those mice bearing primary tumors did not develop secondary tumors [**(B)**, black line], while mice primarily inoculated with irradiated cells developed tumors similar to those of the naïve group [**(B)**, purple and green lines, respectively]. Mean values (*n* = 8) and the SEM are displayed. Time points showing statistical significance when compared to the group of mice implanted with VC are highlighted. Two stars and one star indicate statistical significance at the *p* < 0.01 and *p* < 0.05 levels, respectively. The two way ANOVA test corrected by Bonferroni was applied in this experiment.

Tumor growth of the B16F10 cells was studied in BALB/c mice by implanting four million viable parental B16F10-644 cells s.c. into their right flanks (viability >90%). Five days later, black solid tumors were observed beneath the shaved skin. These tumors reached their maximum size between days 12 and 15 and spontaneously regressed thereafter (Figure 4A). In most cases, small tumors (5–10 mm^3^) persisted subcutaneously in the animal showing no further change in size. Tumor recurrence was verified by keeping mice under observation for more than 2 months; no further tumor growth was seen during this time period. Since the tumors are growing in allogeneic hosts, we regard this as allograft rejection. Metastases were never observed in any organ.

B16F10 is a poorly immunogenic cell line, not able to generate concomitant immunity in its syngeneic host. Accordingly, C57BL/6 mice bearing a progressive tumor are not able to elicit a protective immune response (14). In order to evaluate the immunity in this allogeneic model, mice were implanted s.c. as follows: one group was implanted with 4 million viable cells, the second group with lethally UVB-irradiated cells. A third group was injected with Ringer solution (naïve group). After 12 days, all groups were challenged s.c. with 2 million viable cells in the opposite flank. The black line in Figure 4A shows growth and rejection of primary tumors derived from 4 million viable cells. Mice implanted with UVB-irradiated cells showed no growth of primary tumors (Figure 4A, purple line *p* < 0.05 compared to the black line). After challenge, all mice carrying primary tumors rejected secondary tumors (Figure 4B, black line). In contrast, mice that had received UVB-irradiated cells supported the growth of secondary tumors, as the naïve group did (Figure 4B, purple *p* < 0.05 and green *p* < 0.01 lines compared to the black line). This observation suggests the existence of tumor related immunity in the allogeneic model. We propose to use this system for the evaluation of host immunity after immunization with dead and dying cells.

### Allogeneic response elicited by dying and dead cells

**Figure 5:**
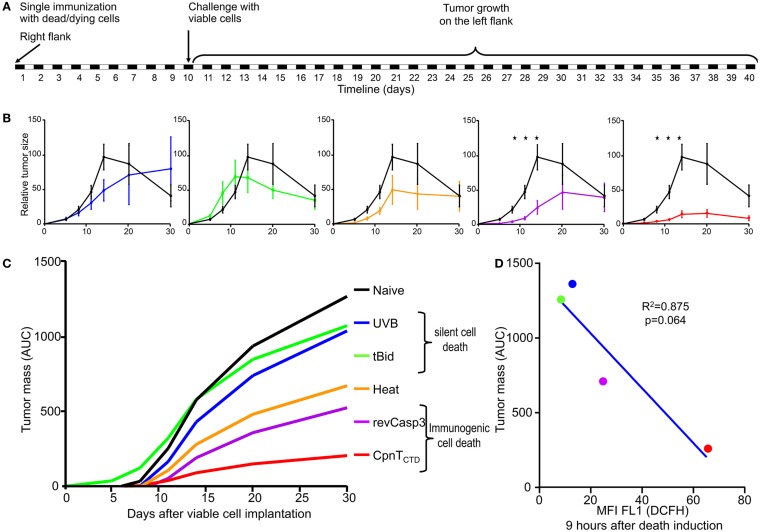
**Immune response against dead or dying allogeneic tumor cells**. BALB/c mice (*n* = 5) were immunized in the right flank s.c. (single dose) with B16F10 dying/dead cells. After 10 days, mice were challenged s.c. in the left flank with 2 million viable cells of the parental cell line B16F10-644. Tumor growth was monitored for 30 further days **(A)**. Cell death was induced by UVB irradiation; heat shock; doxycycline-controlled expression of death proteins tBid, revCasp-3, and CpnT_CTD_. Displayed are the mean values (*n* = 5) of relative tumor volumes and SEM [**(B)**, *p < 0.05 after Mann–Whitney *U* test] and the integral of tumor size [**(C)**, total tumor mass]. Inverse association between ROS production and total tumor mass developed in the allogeneic host **(D)**.

The allogeneic implantation of dead and dying tumor cells in an immune-competent host allows us to evaluate whether the mechanism of cell death induces a silent, a tolerogenic, or an immunogenic cell death by recording the response after a standardized challenge. A silent type of cell death would not affect the growth of the allotumor, a tolerogenic type of cell death would overcome the expected allograft rejection, while an immunogenic cell death would favor the rejection of the allotumor. The host response against each previously characterized form of cell death was assessed in 9–11-week-old female BALB/c (WT) mice immunized with dying/dead B16F10 cells induced to die by the following stimuli: (1) UVB irradiation; (2) doxycycline-controlled expression of the death proteins tBid, revCasp-3, and CpnT_CTD_; and (3) heat shock. Stably transfected B16F10 cells were induced to die by adding 10 μg/ml doxy to the tissue culture dishes (150 cm^2^) at 37°C and 5% CO_2_ for 5 h. Afterward, UVB irradiation (240 mJ/cm^2^) was performed in order to kill the remaining non-responsive cells. Previous experiments had demonstrated that the additional UVB irradiation at this time point neither altered the expression level of the death-inducing proteins nor the kinetics of the specific death phenotype (Figure S1 in Supplementary Material). Sixteen million dying/dead cells were injected s.c. in the right flank as single immunization dose at day 0. After 10 days, mice were challenged in the left flank with 2 million viable cells of the parental cell line B16F10-644. Tumor growth was recorded at days 5, 8, 11, 14, 20, and 30 after challenge (Figure 5A). Viable cells implanted in naïve BALB/c mice generated tumors that reached their maximal size after 2 weeks followed by regression, as expected (Figure 5B, black lines). This group was used as reference cohort.

Mice immunized with UVB-irradiated cells and tBid-expressing cells developed tumors with sizes similar to that of the naïve group. This points to a state of unresponsiveness in these mice (Figure 5B). In contrast, and especially at the days 8, 11, and 14, mice immunized with cells dying because of the expression of revCasp-3 or CpnT_CTD_ displayed significantly smaller tumors than naïve mice (Figure 5B; *p* < 0.05).

In order to estimate the total tumor mass generated for each immunization cohort, an analysis based on the cumulative area under the curve (integral) was performed. Figure 5C shows the total amount of tumor mass developed in the allogeneic host for each cohort. Mice immunized with UVB-irradiated cells or with cells that express tBid developed similar tumor masses. Conversely, mice immunized with dying/dead cells because of revCasp-3 and CpnT_CTD_ expression developed significantly smaller tumor masses (Figure 5C). Based on these observations, we considered UVB irradiation and over-expression of tBid as silent or tolerogenic forms of cell death. Accordingly, over-expression of revCasp-3 and CpnT_CTD_ induced immunogenic cell death. At best, only a weak immunogenic response was elicited by cells treated with heat shock reflecting that the rapid induction of necrosis by heat shock has the inevitable disadvantage of diffusion of danger signals to the medium. One major advantage of doxy-controlled induction of cell death, in particular necrotic cell death, is the achievement of a necrotic phenotype without physical interaction with the cells.

Interestingly, those forms of death showing significant ROS production upon *in vitro* stimulation died in an immunogenic fashion (Figure 5D). To more closely address the role of ROS production in the development of allo-responses, we treated ROS-producing CpnT_CTD_-expressing dead and dying cells with the anti-oxidant NAC before immunization. This treatment resulted in the amelioration of the allo-response against the challenge; however, it was not statistically significant (Figure S2 in Supplementary Material).

## Discussion

Conventional studies characterizing cell death are based on PS exposure and ion selectivity of the plasma membrane (AxA5 and PI staining) (15–17). These methods have traditionally classified cells as viable, apoptotic, or necrotic. However, because of the important role of the mitochondria as modulator of cell death, a concomitant analysis that monitors changes in the mitochondrial membrane potential is required. On the one hand, mitochondria play a role during apoptosis through the release of several apoptogenic proteins located in the inter-membrane space and, consequently, in apoptosome formation (18). On the other hand, mitochondria determine the outcome of many ATP-dependent cell physiological processes and, thus, are important for necrotic cell death. In this work, we used a modified staining and cytofluorometry protocol for a more detailed analysis of tumor cell death induced by various stimuli. Its major advantage is the possibility to classify at least eight different stages of cell death (viable, stressed, early/medium/late apoptosis, early/late secondary necrosis, primary necrosis) in a fast and reliable one-tube assay. A similar method employing a four-color staining for evaluation of PS exposure (AxA5-FITC), plasma membrane integrity (PI), mitochondrial membrane potential (JC-1), and nuclear DNA content (Hoechst 33342) was reported (19). However, a clear classification and identification of different types and stages of cell death was missing in that report.

Usually, standard methods are employed to generate dead cells for immunization experiments, e.g., *in vitro* induction of apoptosis/necrosis in cells and their subsequent injection into the mouse. This approach allowed the dissection of important modulatory effects of apoptotic cells in living multicellular organisms, as well as the employment of adjuvants in cell-based immunization models (20–22). However, the manipulation of dying and dead cells *ex vivo* has the disadvantage of their rapid progression from apoptosis to secondary necrosis and the concomitant decay and dilution of labile and short-range active immunomodulatory signals, respectively. In order to avoid the aforementioned limitations, we have established a conditional doxycycline-dependent expression system able to trigger various types of tumor cell death. Vectors harboring cDNAs encoding for tBid, revCasp-3, and CpnT_CTD_ were stably transfected into B16F10 melanoma cells.

tBid is a major pro-apoptotic protein activated in both extrinsic and intrinsic pathways and is an important connector between these canonical apoptosis pathways. tBid translocates to the mitochondria promoting mitochondrial outer membrane permeabilization (MOMP), a process involving self-assembly of activated BAX and BAK into transmembrane pores, which can be inhibited by anti-apoptotic BCL2 family proteins (23–25). Moreover, tBid accumulation determines the timing of MOMP (26). In addition to cytochrome c, tBid induces the release of further mitochondrial death effectors that promote caspase-independent apoptosis and induce mitochondrial remodeling. All these processes result in loss of the mitochondrial membrane potential, blocking of ATP synthesis and, consequently, loss of function of ATP-dependent transporters.

Over-expression of tBid can be considered to represent a harsh stimulus that quickly drives cells toward advanced stages of the apoptotic process with metabolic collapse leading to an early loss of plasma membrane integrity and rupture. We have defined this particular state of early loss of plasma membrane integrity as early secondary necrosis. It clearly differs from primary necrosis because of its susceptibility to inhibition by caspase inhibitors. Similar stimuli have already been reported to induce necrosis (27–29). This new cell death state may have particular relevance under clinical conditions like treatment with chemotherapy in cancer. Our cell death classification method allowed the identification of a further new stage in the process of dying, which we propose to refer to as *stressed* cells. The latter were observed especially when the over-expression of tBid was induced in the presence of z-VAD-fmk. Stressed cells show low-mitochondrial potential, suggesting the presence of MOMP and severe damage to the mitochondrial membranes in the absence of any further signs of apoptosis. The pan-caspase inhibitor z-VAD-fmk efficiently blocks certain types of apoptosis. In consequence, the common hallmarks of the execution phase of apoptosis, like PS exposure, cell shrinkage, and DNA fragmentation are inhibited. The cells appear viable although they are actually dead because of severe damage of the mitochondrial membranes after over-expression of tBid, which directly activates the mitochondrial pathway.

Compared to the expression of tBid, melanoma cells dying after the expression of revCasp-3 showed slower cell death kinetics. Twenty four hours of expression of revCasp-3 were required to reach a similar degree of cell death. Caspase-3 is the major caspase activated during the execution phase of apoptosis (30). However, expression of revCasp-3 does not directly act on the mitochondria to induce MOMP. Caspase-3 needs to cleave sufficient MOMP-inducing substrates like Bid to tBid before MOMP and cell death can occur by a feed-forward amplification loop (31). Additionally, caspase-3 can then enter the mitochondria and cleave specific substrates of the electron transfer chain, which will ultimately result in mitochondrial uncoupling and loss of membrane potential. Addition of z-VAD-fmk to revCasp-3 expressing cells inhibited up to 95% of the cell death occurring (more than 90% viable cells). These results demonstrate that, after blocking caspases, damage to mitochondria and to cell morphology and physiology was effectively inhibited, as confirmed by the lack of appearance of large numbers of stressed cells in Figure 2C.

Induction of apoptosis by UVB irradiation has been recognized to be a complex process involving a variety of independent pathways. There are at least two major mechanisms involved in apoptosis induced by UVB: (1) DNA damage; UVB induces two types of lesions in chromosomal DNA, photoproducts, and cyclobutane pyrimidine dimers (CPD), the latter being the predominant ones (32). (2) Cell death receptor activation; UVB is able to directly activate cell surface receptors (i.e., CD95/Fas and TNF receptor-1) by inducing receptor trimerization and clustering without ligand interaction (33–35). In our study, B16F10 melanoma cells were lethally irradiated with 240 mJ/cm^2^ UVB applied as a single dose. Lower doses failed to completely kill the cells (data not shown). In the 24 h period following irradiation, we observed slow cell death kinetics mainly characterized by the presence of stressed (mitochondrial damage) and early secondary necrotic cells (membrane disruption and DNA preservation). Substantial amounts of late apoptotic and late secondary necrotic cells were only observed after 48 h.

Cells dying by UVB irradiation in the presence of z-VAD-fmk also showed a significant increase in the fraction of stressed cells, indicating a blockade of the apoptotic execution phase, which may be responsible for the preservation of the cell’s morphology. In the presence of z-VAD-fmk, the percentage of viable cells did not change significantly, showing that inhibition of caspases cannot rescue cells from death. This observation challenges previous own and foreign reports presenting effective inhibition of UVB-induced apoptosis by z-VAD-fmk (36–38). Employing our multiparametric analysis, we here put forward a more detailed characterization of cell death phenotypes induced by several stimuli. We conclude that UVB mainly acts in B16F10 cells by triggering the intrinsic mitochondrial death pathway.

Necrosis is a cellular state that follows acute injuries, sudden anoxia, or extreme stimuli (heat, irradiation, toxins, mechanical, or oxidative stress) and, according to that, it can be viewed as a violent kind of cell death. Conventional methods used to induce necrosis in immunological studies applied heat shock at 56°C or alternating cycles of freeze–thawing. In this work, we present an alternative approach expressing a necrosis inducing protein (CpnT_CTD_) via a doxy-dependent inducible expression system. CpnT is a novel outer membrane protein of *M. tuberculosis* containing a C-terminal domain that is cytotoxic when expressed in eukaryotic cells (8). It has been suggested to be required for the escape of *M. tuberculosis* from macrophages allowing subsequent bacterial dissemination. However, how CpnT_CTD_ induces necrosis is still mechanistically elusive. Six hours after the addition of doxy, a significant fraction of stressed cells was observed. This suggests loss of the mitochondrial membrane potential and mitochondrial damage. Twelve and 18 h after the addition of doxy, more than 50 and 80% of the cells were primary necrotic, respectively. Only after 48 h post-doxy addition, was DNA degradation observed. The decreased DNA content of the latter mimics some features of secondary necrotic death. However, DNA degradation occurred in cells that had already lost their membrane integrity. The cells should, therefore, be referred to as post-necrotic and not as post-apoptotic. The passive influx of DNA degrading enzymes and/or Ca^2+^ most likely causes the chromatin degradation observed in necrotic cells. Expression of the CpnT_CTD_ protein in the presence of z-VAD-fmk showed the same phenotype as those not treated with the inhibitor, arguing for a caspase-independent type of cell death. A classical method to induce necrosis is a heat shock at 56°C for 30 min. As expected, cells became necrotic immediately after heating. This process was also not affected by the presence of z-VAD-fmk, suggesting that caspases were not required for death induced by this kind of heat shock.

Most anti-cancer therapies, like chemotherapy and radiotherapy, aim to induce cancer cell death. However, a central problem is to understand how the immune system determines whether cell death elicits immunogenic, tolerogenic, or silent responses. It has been reported that type and/or phase of cell death affect the immune response (3, 39). For example, cells treated with antracyclines, oxaliplatin, or UVC light develop a kind of endoplasmic reticulum (ER) stress response that involves recruitment of several actors of the apoptotic pathway and contributes to the exposure of calreticulin, which represents an important determinant of immunogenic cell death (40–42).

In order to evaluate whether a certain type of cell death is immunogenic or tolerogenic/silent, an allogeneic murine graft rejection model was employed. The s.c. tumor progression in BALB/c mice was monitored after immunizing the host with various kinds of dying/dead cells. We hypothesized that immunization with tolerogenic or silent forms of cell death would overcome or not affect the natural host immunity allowing tumor development in BALB/c mice, respectively. In contrast, immunogenic cell death would result in faster and more efficient rejection and persistent immunity (3, 43–47). Syngeneic implantation of viable B16F10 cells in C57/Bl6 mice reportedly resulted in high mortality because of the potent carcinogenicity making it difficult to evaluate the immune response against various kinds of cell death (48, 49). The allogeneic model, instead, allows us to compare the immune modulatory effects of cell death forms without compromising the welfare of the host (10–12).

Immune responses elicited by dying/dead cells in BALB/c mice were dependent on the death stimulus applied to the vaccine. Mice implanted with viable melanoma cells transiently produced primary tumors, but never developed secondary tumors after re-challenge (100% rejection). In contrast, mice immunized with irradiated cells supported the growth of secondary tumors similar to the naïve cohort. In order to explore this effect thoroughly, mice were challenged after having been immunized with dying/dead cells killed by various mechanisms.

Expression of the necrosis inducing protein domain CpnT_CTD_ in a B16F10 cell-based vaccine resulted in the most immunogenic type of cell death. Unexpectedly, expression of the pro-apoptotic constitutively active form of caspase-3 (revCasp-3) also induced a strongly immunogenic type of cell death. Massive apoptosis induced by the regulated expression of revCasp-3 in already implanted syngeneic tumors has been reported to be immunogenic (50). Expression of caspase-3 induced a high percentage of necrotic cell death (early and late secondary necrosis). Heat-necrotized cells, which display membrane disruption already before injection, exhibited poor immunogenicity. This points to short-lived necrotic cell-derived signaling molecules, which would not be active anymore in the vaccine that had been necrotized before injection. Summarizing, in our model the time of appearance of membrane disruption in B16F10 cells did not correlate with the type of immune response the cells elicited. Noteworthy, UVB-irradiated cells showed the lowest percentage of necrotic cells and failed to trigger immunity. tBid-expressing cells and UVB-irradiated cells used for immunization clearly behaved silently and allowed the growth of allogeneic tumors, despite the presence of high levels of necrotic cells, as in naïve animals.

The unresponsiveness observed in our system cannot be explained solely by the exposure of PS. Similarly, DAMPs released or expressed after disruption of the plasma membrane are also not sufficient to explain the immunogenicity observed in our model. Therefore, we looked for common features of the two “silent” types of cell death, over-expression of tBid and UVB irradiation, and of the two immunogenic types of cell death, over-expression of revCasp-3 and CpnT_CTD_. The common denominator in both cases is the prominent role of the mitochondria in the execution of death (39).

In our system, activation of the intrinsic pathway either by the recombinant expression of cleaved active Bid or by UVB irradiation may have caused death without relevant ROS production during the time frame the cells were employed for immunization (3–9 h after doxy treatment). Although some caspase-3 activity is to be expected in both tBid- and UVB-induced death, this was not enough to secondarily generate ROS. In contrast, downstream expression of revCasp-3 and CpnT_CTD_ may have caused death by activation of terminal effector mechanisms excluding the involvement of mitochondria. Therefore, we assume that in the revCasp-3 and CpnT_CTD_ cases, execution of cell death starts before mitochondrial function is severely compromised. After over-expression of revCasp-3, MOMP formation is induced allowing access of caspase-3 to the mitochondrial inter-membrane space, cleaving specific substrates in the complex I of the electron transport chain (like p75 NDUSF1) (51, 52). As a consequence, the electron transport is disrupted, and ROS is generated. In contrast, tBid expression causes severe damage to the mitochondria measured as a fast loss of the mitochondrial potential, suggesting fast MOMP formation thereby destroying electron transport abruptly. We propose that ROS can be produced in cells overexpressing revCasp-3 and possibly CpnT_CTD_ because mitochondrial activity is not severely affected by an intrinsic death stimulus. Ongoing cell death in the presence of functioning mitochondria would be important for intracellular ROS accumulation. Free radicals generated in this context may induce considerable stress in the ER, which has been associated with immunogenicity of the dying cells (3).

We suggest that intracellular accumulation of ROS intermediates may play a critical role in the generation of DAMPs affecting the ongoing allogeneic immune response. For example, oxidative damage to DNA enhances its immune-stimulatory capabilities once processed by the immune system (53). Avoiding intracellular accumulation of ROS by a selective manipulation of mitochondrial functionality before killing cells is a possible option to down-regulate the subsequent immune response. Reversely, conserving mitochondrial function as long as possible during cancer cell killing may increase the immunogenicity, if this is wanted.

Although NAC significantly inhibited ROS production in CpnT_CTD_-expressing cells *in vitro* (Figure 3), the employment of this anti-oxidant during cell death induced by the expression of CpnT_CTD_ was not sufficient to abrogate their immunogenicity in our allo-immunization model. Interestingly, the addition of the specific mitochondrial ROS scavenger mitoTEMPO induced significant inhibition of ROS production in B16F10-revCasp-3 and B16F10-CpnT_CTD_ dying cells *in vitro* arguing for an important role of the mitochondria in ROS production. Since inhibition *in vitro* by NAC or mitoTEMPO was partial, ROS may be additionally produced in other cellular compartments like the ER. Noteworthy, it was demonstrated in yeasts that mitochondrial dysfunction indirectly induces ROS production in the ER by a novel mechanism mediated by suppression of the endoplasmic reticulum-associated degradation (ERAD) pathway (54, 55). In line with this, ER stress may result in increased cytoplasmic Ca^2+^ concentrations and immunogenic cell death (56). Further investigation addressing the source and localization of ROS in these cells is needed to elucidate the exact role of ROS production during cell death and its immunological consequences, especially in *in vivo* models.

We speculate that some of the mechanisms observed in this manuscript might also work in patients with tumors, during tumor therapy and in some syngeneic tumor models. However, the application of tumor vaccines in human beings has, so far, been less successful. The association between intracellular ROS accumulation in dying cells and allogeneic tumor rejection may have the potential to intentionally shape anti-tumor immunity.

## Conflict of Interest Statement

The authors declare that the research was conducted in the absence of any commercial or financial relationships that could be construed as a potential conflict of interest.

## Supplementary Material

The Supplementary Material for this article can be found online at http://journal.frontiersin.org/Journal/10.3389/fimmu.2014.00560/abstract

Figure S1 **UVB irradiation of B16F10 cells after doxycycline treatment**. In order to rule out that an additional irradiation step interferes with the type of cell death induced by specific expression of the respective cytotoxic protein in the cell lines B16F10-tBid, B16F10-revCasp-3, and B16F10-CpnT_CTD_, cells were induced to die with doxycyline (5 μg/ml) and irradiated with a single dose of 240 mJ/cm^2^ UVB at different time points (*t* = 4 h, *t* = 5 h, *t* = 6 h, and *t* = 7 h). Eighteen hours after doxycycline addition (*t*_0_), the cells were harvested by trypsinization and analyzed by FACS for PI staining. Cells killed solely by UVB or doxycycline were used as controls. Blue bars show death induced by irradiation alone. Red bars show death by doxycycline-regulated expression of each cytotoxic protein alone. Green bars show the combined effect of cytotoxic protein expression plus irradiation at different time points. Note that cells irradiated 4, 5, 6, and 7 h after doxycycline addition (green bars) died to the same extent as cells killed by doxycycline alone (red bars).These results suggest that after 4 h of incubation with doxy, the additional irradiation step did not significantly affect the degree of doxycycline-induced cell death despite of the additional damage caused by the irradiation. We, therefore, assumed that after 4 h the cytotoxic protein expression is sufficient to induce cell death as it would happen without irradiation. Irradiation at earlier time points significantly impaired doxycycline-regulated cell death (data not shown). This procedure ensured killing of the doxycycline-resistant cells in primary grafts in order to consequently avoid complications caused by proliferative signals emitted from the dying cells acting on the surviving tumor cells.

Figure S2 **Immune response against allogeneic dead and dying cells in the presence of the ROS inhibitor NAC**. BALB/c mice were immunized in the right flank s.c. (single dose) with dying B16F10 cells expressing CpnT_CTD_ (red lines/bars) or dying B16F10 cells expressing CpnT_CTD_ in the presence of the ROS inhibitor *n*-Acetylcysteine (NAC, green lines/bars). Displayed are the mean values (*n* = 7) of the relative tumor volumes and their SEMs and the respective integral of tumor size (total tumor mass) that was obtained in mice challenged s.c. in the left flank with 2 million VC of the parental cell line B16F10-644 10 days after immunization. The Mann–Whitney *U* test was applied to compare groups.

## References

[1] Matzinger P. The danger model: a renewed sense of self. Science (2002) 296(5566):301–5. 10.1126/science.1071059 11951032

[2] Schroppel B Legendre C. Delayed kidney graft function: from mechanism to translation. Kidney Int (2014) 86:251–8. 10.1038/ki.2014.18 24522494

[3] Green DR Ferguson T Zitvogel L Kroemer G. Immunogenic and tolerogenic cell death. Nat Rev Immunol (2009) 9(5):353–63. 10.1038/nri2545 19365408 PMC2818721

[4] Kepp O Senovilla L Kroemer G Immunogenic cell death inducers as anticancer agents. Oncotarget (2014) 5(14):5190–1. 25114034 10.18632/oncotarget.2266 PMC4170601

[5] Kelland LR. Of mice and men: values and liabilities of the athymic nude mouse model in anticancer drug development. Eur J Cancer (2004) 40(6):827–36. 10.1016/j.ejca.2003.11.028 15120038

[6] Kepp O Galluzzi L Martins I Schlemmer F Adjemian S Michaud M Molecular determinants of immunogenic cell death elicited by anticancer chemotherapy. Cancer Metastasis Rev (2011) 30(1):61–9. 10.1007/s10555-011-9273-4 21249425

[7] Maueroder C Chaurio RA Platzer S Munoz LE Berens C. Model systems for rapid and slow induction of apoptosis obtained by inducible expression of pro-apoptotic proteins. Autoimmunity (2013) 46(5):329–35. 10.3109/08916934.2012.752463 23173725

[8] Danilchanka O Sun J Pavlenok M Maueröder C Speer A Siroy A An outer membrane channel protein of *Mycobacterium tuberculosis* with exotoxin activity. Proc Natl Acad Sci U S A (2014) 111:6750–5. 10.1073/pnas.1400136111 24753609 PMC4020113

[9] Munoz LE Maueroder C Chaurio R Berens C Herrmann M Janko C. Colourful death: six-parameter classification of cell death by flow cytometry – dead cells tell tales. Autoimmunity (2013) 46(5):336–41. 10.3109/08916934.2012.755960 23231469

[10] United Kingdom Co-ordinating Committee on Cancer Research (UKCCCR) guidelines for the welfare of animals in experimental neoplasia (second edition). Br J Cancer (1998) 77(1):1–10 10.1038/bjc.1998.1 PMC2151254 9459138

[11] Jones-Bolin S. Guidelines for the care and use of laboratory animals in biomedical research. Curr Protoc Pharmacol (2012) 59:4B:A.4B.1–9. 10.1002/0471141755.pha04bs59 23258596

[12] Vale C Stewart L Tierney J. Trends in UK cancer trials: results from the UK coordinating committee for cancer research national register of cancer trials. Br J Cancer (2005) 92(5):811–4. 10.1038/sj.bjc.6602425 15756251 PMC2361907

[13] Janko C Munoz L Chaurio R Maueroder C Berens C Lauber K Navigation to the graveyard-induction of various pathways of necrosis and their classification by flow cytometry. Methods Mol Biol (2013) 1004:3–15. 10.1007/978-1-62703-383-1_1 23733565

[14] Turk MJ Guevara-Patino JA Rizzuto GA Engelhorn ME Sakaguchi S Houghton AN. Concomitant tumor immunity to a poorly immunogenic melanoma is prevented by regulatory T cells. J Exp Med (2004) 200(6):771–82. 10.1084/jem.20041130 15381730 PMC2211964

[15] Appelt U Sheriff A Gaipl US Kalden JR Voll RE Herrmann M Viable, apoptotic and necrotic monocytes expose phosphatidylserine: cooperative binding of the ligand annexin V to dying but not viable cells and implications for PS-dependent clearance. Cell Death Differ (2005) 12(2):194–6 10.1038/sj.cdd.4401527 15540112

[16] Franz S Frey B Sheriff A Gaipl US Beer A Voll RE Lectins detect changes of the glycosylation status of plasma membrane constituents during late apoptosis. Cytometry A (2006) 69(4):230–9 10.1002/cyto.a.20206 16498674

[17] Zhang G Gurtu V Kain SR Yan G. Early detection of apoptosis using a fluorescent conjugate of annexin V. Biotechniques (1997) 23(3):525–31. 9298227 10.2144/97233pf01

[18] Saelens X Festjens N Vande Walle L van Gurp M van Loo G Vandenabeele P. Toxic proteins released from mitochondria in cell death. Oncogene (2004) 23(16):2861–74. 10.1038/sj.onc.1207523 15077149

[19] Troiano L Ferraresi R Lugli E Nemes E Roat E Nasi M Multiparametric analysis of cells with different mitochondrial membrane potential during apoptosis by polychromatic flow cytometry. Nat Protoc (2007) 2(11):2719–27. 10.1038/nprot.2007.405 18007607

[20] Frey B Schildkopf P Rodel F Weiss EM Munoz LE Herrmann M AnnexinA5 renders dead tumor cells immunogenic – implications for multimodal cancer therapies. J Immunotoxicol (2009) 6(4):209–16. 10.3109/15476910903204058 19908939

[21] Gaipl US Munoz LE Rodel F Pausch F Frey B Brachvogel B Modulation of the immune system by dying cells and the phosphatidylserine-ligand annexin A5. Autoimmunity (2007) 40(4):254–9. 10.1080/08916930701357331 17516206

[22] Munoz LE Frey B Pausch F Baum W Mueller RB Brachvogel B The role of annexin A5 in the modulation of the immune response against dying and dead cells. Curr Med Chem (2007) 14(3):271–7. 10.2174/092986707779941131 17305532

[23] Li H Zhu H Xu CJ Yuan J. Cleavage of BID by caspase 8 mediates the mitochondrial damage in the Fas pathway of apoptosis. Cell (1998) 94(4):491–501. 10.1016/S0092-8674(00)81590-1 9727492

[24] Luo X Budihardjo I Zou H Slaughter C Wang X. Bid, a Bcl2 interacting protein, mediates cytochrome c release from mitochondria in response to activation of cell surface death receptors. Cell (1998) 94(4):481–90. 10.1016/S0092-8674(00)81589-5 9727491

[25] Youle RJ Strasser A. The BCL-2 protein family: opposing activities that mediate cell death. Nat Rev Mol cell Biol (2008) 9(1):47–59. 10.1038/nrm2308 18097445

[26] Spencer SL Gaudet S Albeck JG Burke JM Sorger PK. Non-genetic origins of cell-to-cell variability in TRAIL-induced apoptosis. Nature (2009) 459(7245):428–32. 10.1038/nature08012 19363473 PMC2858974

[27] Eastman A. Apoptosis: a product of programmed and unprogrammed cell death. Toxicol Appl Pharmacol (1993) 121(1):160–4. 10.1006/taap.1993.1141 8337697

[28] Lieberthal W Triaca V Levine J. Mechanisms of death induced by cisplatin in proximal tubular epithelial cells: apoptosis vs. necrosis. Am J Physiol (1996) 270(4 Pt 2):F700–8. 8967349 10.1152/ajprenal.1996.270.4.F700

[29] Martin SJ Green DR Protease activation during apoptosis: death by a thousand cuts? Cell (1995) 82(3):349–52 10.1016/0092-8674(95)90422-0 7634323

[30] Elmore S Apoptosis: a review of programmed cell death. Toxicol Pathol (2007) 35(4):495–516 10.1080/01926230701320337 17562483 PMC2117903

[31] Shelton SN Shawgo ME Robertson JD. Cleavage of Bid by executioner caspases mediates feed forward amplification of mitochondrial outer membrane permeabilization during genotoxic stress-induced apoptosis in Jurkat cells. J Biol Chem (2009) 284(17):11247–55. 10.1074/jbc.M809392200 19233849 PMC2670129

[32] Patrick MH Studies on thymine-derived UV photoproducts in DNA – I. Formation and biological role of pyrimidine adducts in DNA. Photochem Photobiol (1977) 25(4):357–72 10.1111/j.1751-1097.1977.tb07355.x 882597

[33] Aragane Y Kulms D Metze D Wilkes G Poppelmann B Luger TA Ultraviolet light induces apoptosis via direct activation of CD95 (Fas/APO-1) independently of its ligand CD95L. J Cell Biol (1998) 140(1):171–82. 10.1083/jcb.140.1.171 9425165 PMC2132609

[34] Rehemtulla A Hamilton CA Chinnaiyan AM Dixit VM. Ultraviolet radiation-induced apoptosis is mediated by activation of CD-95 (Fas/APO-1). J Biol Chem (1997) 272(41):25783–6. 10.1074/jbc.272.41.25783 9325306

[35] Rosette C Karin M. Ultraviolet light and osmotic stress: activation of the JNK cascade through multiple growth factor and cytokine receptors. Science (1996) 274(5290):1194–7. 10.1126/science.274.5290.1194 8895468

[36] Garcia-Calvo M Peterson EP Leiting B Ruel R Nicholson DW Thornberry NA. Inhibition of human caspases by peptide-based and macromolecular inhibitors. J Biol Chem (1998) 273(49):32608–13. 10.1074/jbc.273.49.32608 9829999

[37] Kulms D Zeise E Poppelmann B Schwarz T. DNA damage, death receptor activation and reactive oxygen species contribute to ultraviolet radiation-induced apoptosis in an essential and independent way. Oncogene (2002) 21(38):5844–51. 10.1038/sj.onc.1205743 12185583

[38] Schiller M Blank N Heyder P Herrmann M Gaipl US Kalden JR Induction of apoptosis by spermine-metabolites in primary human blood cells and various tumor cell lines. Apoptosis (2005) 10(5):1151–62. 10.1007/s10495-005-1188-5 16151648

[39] Galluzzi L Vitale I Abrams JM Alnemri ES Baehrecke EH Blagosklonny MV Molecular definitions of cell death subroutines: recommendations of the nomenclature committee on cell death 2012. Cell Death Differ (2012) 19(1):107–20. 10.1038/cdd.2011.96 21760595 PMC3252826

[40] Casares N Pequignot MO Tesniere A Ghiringhelli F Roux S Chaput N Caspase-dependent immunogenicity of doxorubicin-induced tumor cell death. J Exp Med (2005) 202(12):1691–701. 10.1084/jem.20050915 16365148 PMC2212968

[41] Panaretakis T Kepp O Brockmeier U Tesniere A Bjorklund AC Chapman DC Mechanisms of pre-apoptotic calreticulin exposure in immunogenic cell death. EMBO J (2009) 28(5):578–90. 10.1038/emboj.2009.1 19165151 PMC2657583

[42] Zitvogel L Kepp O Senovilla L Menger L Chaput N Kroemer G. Immunogenic tumor cell death for optimal anticancer therapy: the calreticulin exposure pathway. Clin Cancer Res (2010) 16(12):3100–4. 10.1158/1078-0432.CCR-09-2891 20421432

[43] Kepp O Tesniere A Schlemmer F Michaud M Senovilla L Zitvogel L Immunogenic cell death modalities and their impact on cancer treatment. Apoptosis (2009) 14(4):364–75 10.1007/s10495-008-0303-9 19145485

[44] Kepp O Tesniere A Zitvogel L Kroemer G The immunogenicity of tumor cell death. Curr Opin Oncol (2009) 21(1):71–6 10.1097/CCO.0b013e32831bc375 19125021

[45] Kono H Rock KL. How dying cells alert the immune system to danger. Nat Rev Immunol (2008) 8(4):279–89. 10.1038/nri2215 18340345 PMC2763408

[46] Krysko DV Garg AD Kaczmarek A Krysko O Agostinis P Vandenabeele P. Immunogenic cell death and DAMPs in cancer therapy. Nat Rev Cancer (2012) 12(12):860–75. 10.1038/nrc3380 23151605

[47] LaLadoire S Hannani D Vetizou M Locher C Aymeric L Apetoh L Cell-death-associated molecular patterns as determinants of cancer immunogenicity. Antioxid Redox Signal (2013) 20(7):1098–116. 10.1089/ars.2012.5133 23394620

[48] Briles EB Kornfeld S. Isolation and metastatic properties of detachment variants of B16 melanoma cells. J Natl Cancer Inst (1978) 60(6):1217–22. 418183 10.1093/jnci/60.6.1217

[49] Fidler IJ. Biological behavior of malignant melanoma cells correlated to their survival in vivo. Cancer Res (1975) 35(1):218–24. 1109790

[50] Melis MH Simpson KL Dovedi SJ Welman A MacFarlane M Dive C Sustained tumour eradication after induced caspase-3 activation and synchronous tumour apoptosis requires an intact host immune response. Cell Death Differ (2013) 20(5):765–73. 10.1038/cdd.2013.8 23412345 PMC3619244

[51] Ricci JE Gottlieb RA Green DR. Caspase-mediated loss of mitochondrial function and generation of reactive oxygen species during apoptosis. J Cell Biol (2003) 160(1):65–75. 10.1083/jcb.200208089 12515825 PMC2172744

[52] Ricci JE Munoz-Pinedo C Fitzgerald P Bailly-Maitre B Perkins GA Yadava N Disruption of mitochondrial function during apoptosis is mediated by caspase cleavage of the p75 subunit of complex I of the electron transport chain. Cell (2004) 117(6):773–86. 10.1016/j.cell.2004.05.008 15186778

[53] Gehrke N Mertens C Zillinger T Wenzel J Bald T Zahn S Oxidative damage of DNA confers resistance to cytosolic nuclease TREX1 degradation and potentiates STING-dependent immune sensing. Immunity (2013) 39(3):482–95. 10.1016/j.immuni.2013.08.004 23993650

[54] Leadsham JE Sanders G Giannaki S Bastow EL Hutton R Naeimi WR Loss of cytochrome c oxidase promotes RAS-dependent ROS production from the ER resident NADPH oxidase, Yno1p, in yeast. Cell Metab (2013) 18(2):279–86. 10.1016/j.cmet.2013.07.005 23931758

[55] Murphy MP. Mitochondrial dysfunction indirectly elevates ROS production by the endoplasmic reticulum. Cell Metab (2013) 18(2):145–6. 10.1016/j.cmet.2013.07.006 23931748

[56] Kepp O Menger L Vacchelli E Locher C Adjemian S Yamazaki T Crosstalk between ER stress and immunogenic cell death. Cytokine Growth Factor Rev (2013) 24(4):311–8. 10.1016/j.cytogfr.2013.05.001 23787159

