# Supplementary figures and images for: The Progression of Cell Death Affects the Rejection of Allogeneic Tumors in Immune-Competent Mice – Implications for Cancer Therapy
Source: Front Immunol. 2014 Nov 11;5:560. doi: 10.3389/fimmu.2014.00560 (PMC4227513; doi:10.3389/fimmu.2014.00560)

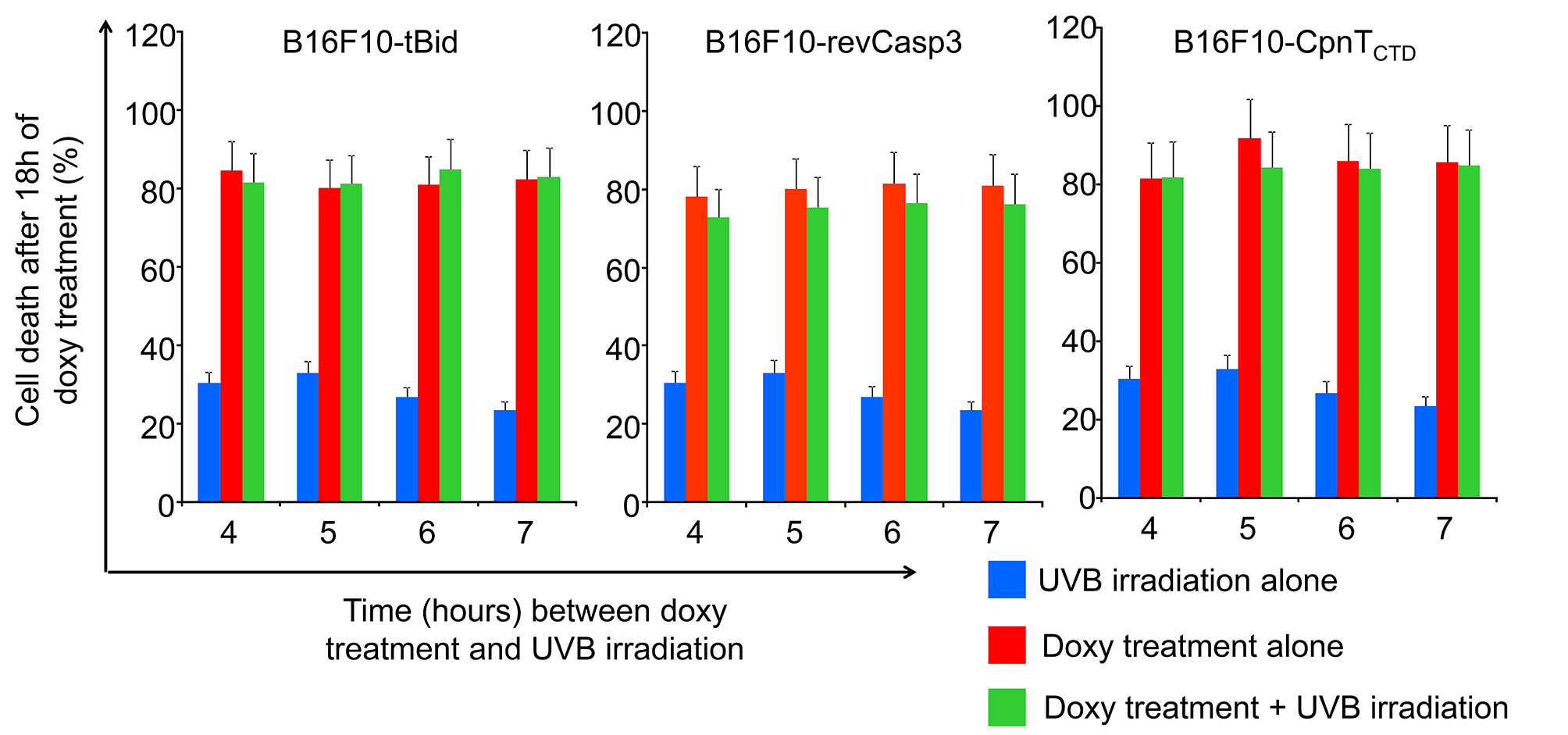

Supplement: Figure S1 — UVB irradiation of B16F10 cells after doxycycline treatment. In order to rule out that an additional irradiation step interferes with the type of cell death induced by specific expression of the respective cytotoxic protein in the cell lines B16F10-tBid, B16F10-revCasp-3, and B16F10-CpnTCTD, cells were induced to die with doxycyline (5 μg/ml) and irradiated with a single dose of 240 mJ/cm2 UVB at different time points (t = 4 h, t = 5 h, t = 6 h, and t = 7 h). Eighteen hours after doxycycline addition (t0), the cells were harvested by trypsinization and analyzed by FACS for PI staining. Cells killed solely by UVB or doxycycline were used as controls. Blue bars show death induced by irradiation alone. Red bars show death by doxycycline-regulated expression of each cytotoxic protein alone. Green bars show the combined effect of cytotoxic protein expression plus irradiation at different time points. Note that cells irradiated 4, 5, 6, and 7 h after doxycycline addition (green bars) died to the same extent as cells killed by doxycycline alone (red bars).These results suggest that after 4 h of incubation with doxy, the additional irradiation step did not significantly affect the degree of doxycycline-induced cell death despite of the additional damage caused by the irradiation. We, therefore, assumed that after 4 h the cytotoxic protein expression is sufficient to induce cell death as it would happen without irradiation. Irradiation at earlier time points significantly impaired doxycycline-regulated cell death (data not shown). This procedure ensured killing of the doxycycline-resistant cells in primary grafts in order to consequently avoid complications caused by proliferative signals emitted from the dying cells acting on the surviving tumor cells. [file Image1.TIF]

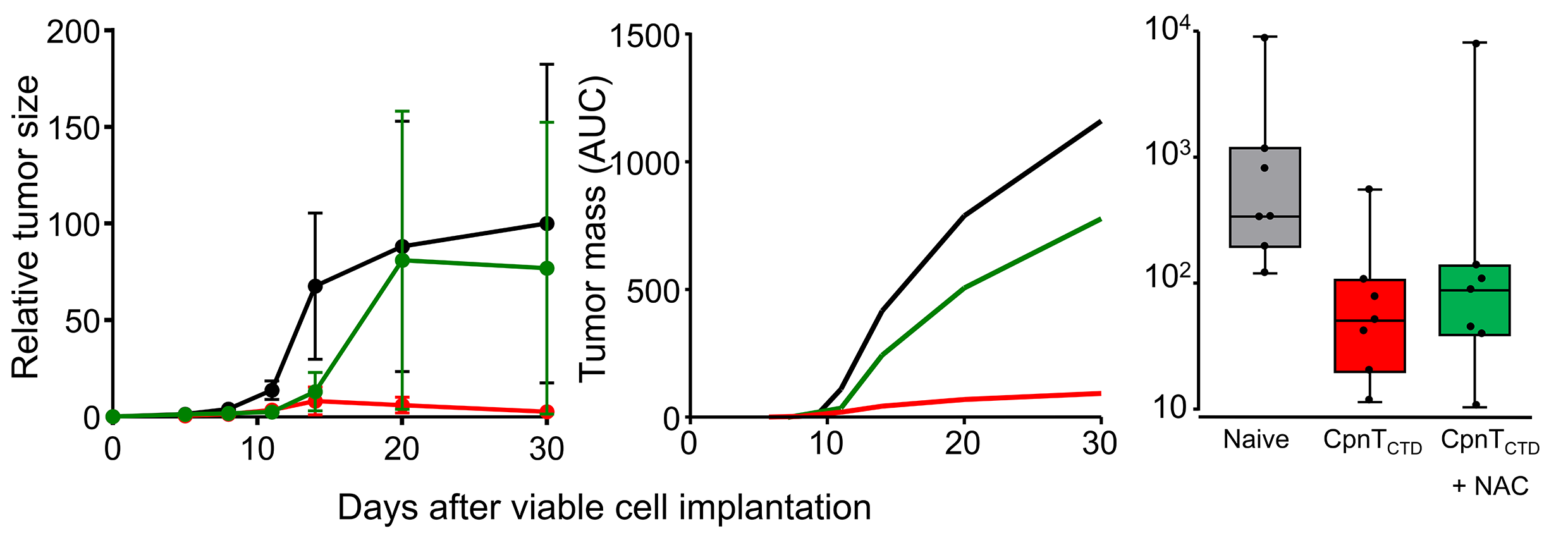

Supplement: Figure S2 — Immune response against allogeneic dead and dying cells in the presence of the ROS inhibitor NAC. BALB/c mice were immunized in the right flank s.c. (single dose) with dying B16F10 cells expressing CpnTCTD (red lines/bars) or dying B16F10 cells expressing CpnTCTD in the presence of the ROS inhibitor n-Acetylcysteine (NAC, green lines/bars). Displayed are the mean values (n = 7) of the relative tumor volumes and their SEMs and the respective integral of tumor size (total tumor mass) that was obtained in mice challenged s.c. in the left flank with 2 million VC of the parental cell line B16F10-644 10 days after immunization. The Mann–Whitney U test was applied to compare groups. [file Image2.TIF]
